# Serum osteoprotegerin is associated with pulse pressure in kidney transplant recipients

**DOI:** 10.1038/srep14518

**Published:** 2015-10-13

**Authors:** Zsofia K. Nemeth, Nicoleta G. Mardare, Maria E. Czira, Gyorgy Deak, Istvan Kiss, Zoltan Mathe, Adam Remport, Akos Ujszaszi, Adrian Covic, Miklos Z. Molnar, Istvan Mucsi

**Affiliations:** 1 3rd Dept. of Internal Medicine and Nephrology, Uzsoki Hospital, Uzsoki u. 29-41, H-1145 Budapest, Hungary; 2 Dialysis and Transplantation Centre, “Dr. C.I. Parhon” University Hospital, 50 Carol 1st Blvd, Iasi 6600, Romania; 3 Institute of Epidemiology and Social Medicine, University of Muenster, Albert-Schweitzer-Campus 1, Gebaude D3, D-48149 Muenster, Germany; 4 Dept. of Nephrology-Hypertension, Szent Imre Teaching Hospital, Tétényi út 12-16, H-1115 Budapest, Hungary; 5 Division of Geriatrics, Semmelweis University Budapest, Hungary; 6 1st Dialysis Centre, B.Braun Avitum Hungary Dialysis Network, Budapest, Hungary; 7 Dept. of Transplantation and Surgery, Semmelweis University, Baross u. 23-26, H-1082 Budapest, Hungary; 8 Institute of Pathophysiology, Semmelweis University, Nagyvárad tér 4., H-1089 Budapest, Hungary; 9 Nephrology Department, University of Medicine and Pharmacy “Gr. T. Popa”, 50 Carol 1st Blvd, Iasi 6600, Romania; 10 Division of Nephrology, Department of Medicine, University of Tennessee Health Science Center, 956 Court Ave, Suite B216B, Memphis, TN, 38163, USA; 11 Department of Medicine, Division of Nephrology, University Health Network, University of Toronto, 585 University Avenue, Toronto, ON, M5G 2N2, Canada

## Abstract

Pulse pressure (PP) reflects increased large artery stiffness, which is caused, in part, by arterial calcification in patients with chronic kidney disease. PP has been shown to predict both cardiovascular and cerebrovascular events in various patient populations, including kidney transplant (KTX) recipients. Osteoprotegerin (OPG) is a marker and regulator of arterial calcification, and it is related to cardiovascular survival in hemodialysis patients. Here we tested the hypothesis that OPG is associated with increased pulse pressure. We cross-sectionally analyzed the association between serum OPG and PP in a prevalent cohort of 969 KTX patients (mean age: 51 +/− 13 years, 57% male, 21% diabetics, mean eGFR 51 +/− 20 ml/min/1.73 m2). Independent associations were tested in a linear regression model adjusted for multiple covariables. PP was positively correlated with serum OPG (rho = 0.284, p < 0.001). Additionally, a positive correlation was seen between PP versus age (r = 0.358, p < 0.001), the Charlson Comorbidity Index (r = 0.232, p < 0.001), serum glucose (r = 0.172, p < 0.001), BMI (r = 0.133, p = 0.001) and serum cholesterol (r = 0.094, p = 0.003). PP was negatively correlated with serum Ca, albumin and eGFR. The association between PP and OPG remained significant after adjusting for multiple potentially relevant covariables (beta = 0.143, p < 0.001). We conclude that serum OPG is independently associated with pulse pressure in kidney transplant recipients.

Cardiovascular (CV) disease is the leading cause of death in kidney transplant (KTX) recipients^1^,^2^. Factors related to mineral and bone disorders (MBD) are important novel cardiovascular risk factors among patients with chronic kidney disease (CKD)^3^,^4^,^5^. MBD is thought to contribute to accelerated vascular calcification in these patients^6^,^7^. Although disorders of bone and mineral metabolism improve after KTX, they remain frequent in kidney transplant recipients^8^ and may contribute to increased CV risk.

Accelerated age-related arterial remodeling and also vascular calcification lead to the loss of large and small vessel distensibility in patients with CKD and end stage kidney disease (ESKD). These changes manifest in increased vascular stiffness, frequently assessed by pulse wave velocity (PWV)^9^. Increased vascular stiffness contributes to profound changes in circulatory function, including an increase in systolic blood pressure (SBP) and widening of pulse pressure (PP)^10^. Pulse pressure (PP), easily available from blood pressure (BP) determination in everyday clinical practice, is considered a surrogate for arterial stiffness^11^. PP independently predicted the risk of death in a large cohort of hemodialysis (HD) patients^12^.

Most^13^,^14^ but not all^15^ studies reported that KTX improves vascular stiffness. Impaired graft function^16^ or new onset diabetes^17^, however, were associated with increased stiffness in this patient population. Vascular stiffness and calcification has been repeatedly shown to predict poor clinical outcome after KTX^18^,^19^. Increased PP is also an independent predictor of cardiovascular events and premature cardiovascular mortality in KTX recipients^20^. The link between PP and clinical outcome is likely due to, at least in part, vascular stiffness, similarly to the link between PP and kidney function^21^. Importantly PP is associated with mineral metabolism abnormalities^10^ suggesting a link between MBD and vascular stiffness and CV disease.

Studies have indicated a role for osteoprotegerin (OPG), a soluble decoy receptor of the osteoclast activator RANKL, in arterial calcification and atherosclerosis^22^,^23^,^24^. Elevated OPG levels were associated with faster progression of vascular calcifications^25^, with PWV and mortality in patients on maintenance dialysis^26^,^27^,^28^ and with CKD^29^,^30^.

Studies in kidney transplant recipients revealed that serum OPG declined during the first 2 weeks after KTX and decreased further during the next 2 weeks^31^. Nonetheless, post-transplant OPG levels predicted coronary^32^ and aortic^33^ calcifications and also mortality^34^,^35^ in KTX patients.

In this cross-sectional prevalent cohort study we tested the hypothesis that elevated serum OPG is associated with increased PP, an easily available surrogate of arterial stiffness and vascular calcification, independent of potential confounding variables in kidney transplant recipients.

## Methods

### Patient population and data collection

All prevalent kidney transplant recipients 18 years of age or older (n = 1214) followed at a single transplant clinic at Semmelweis University in Budapest, Hungary were invited to participate in this observational cohort study. Exclusion criteria were acute rejection within the last 4 weeks, current hospitalization, transplantation in the previous 3 months, acute infection or active bleeding. Of the 993 subjects enrolled, 982 (99%) had baseline blood samples that were available for osteoprotegerin (OPG) assay. All subjects were recruited and baseline assessments were conducted between February 2007 and August 2007 (Malnutrition-Inflammation in Transplant-Hungary Study; MINIT-HU Study). The study methods have been previously described^36^.

### Ethical approval

The study was approved by the Ethics Committee of the Semmelweis University (49/2006). Before enrolment, patients received detailed verbal and written information about the aims and protocol of the study and signed informed consent. All experiments were performed in accordance with relevant guidelines and regulations.

### Laboratory data

Routinely available laboratory data were extracted from the patients’ charts and from the hospital’s electronic laboratory database. Estimated glomerular filtration rate (eGFR) was calculated using the Chronic Kidney Disease Epidemiology Collaboration (CKD-EPI) equation^37^. The following laboratory parameters were tabulated: hemoglobin (Hb), serum C-reactive protein (CRP), total cholesterol, HDL- and LDL-cholesterol, triglyceride, calcium, phosphorus, total alkaline phosphatase, albumin, creatinine and blood urea nitrogen (BUN). Serum intact parathyroid hormone (PTH) (pg/ml) was determined by second-generation electrochemiluminescence assay (iPTH Elecsys System; Roche, Mannheim, Germany).

Serum samples were also collected at the time of the baseline assessment and stored at −70 ^°^C for future use. Serum osteoprotegerin was measured with an immunoassay kits based on a solid-phase sandwich enzyme-linked immunosorbent assay (ELISA) (Biomedica, Vienna, Austria).

### Co-morbidities and blood pressure measurement

We used the modified Charlson Comorbidity Index (CCI)^38^, which is a weighted scoring system based on the presence or absence of each of 17 variables. Earlier it has been reported that the CCI was a predictor of survival in kidney transplanted patients^39^. Each patient enrolled in this study underwent a physical examination and subjective global assessments (SGA) (in order to determine the Malnutrition-Inflammation Score) by a physician (ME Czira) at the baseline visit. Blood pressure was recorded as part of this exam. One calibrated mercury sphygmomanometer was used for each participant. Blood pressure was recorded as the average of three readings after ten minutes rest during the baseline clinic visit.

### Immunosuppressive therapy

Standard maintenance immunosuppressive therapy at our institution consisted of prednisolone, either cyclosporine A microemulsion formulation (CsA) or tacrolimus, combined with mycophenolate-mofetil (MMF) or azathioprine or sirolimus.

### Statistical analysis

We used descriptive statistics to compare clinical and biochemical characteristics across tertiles of baseline serum OPG. Data were summarized using proportions, means (±standard deviation, SD) or medians (interquartile range, IQR) as appropriate. Normality of the distribution of continuous variables was checked with the Kolmogorov-Smirnov test. Variables with skewed distribution were natural log-transformed for all analyses. Correlation between serum OPG and PP versus continuous variables was tested with Pearson correlation analysis. To compare variables between tertiles of serum OPG, one-way analysis of variance (ANOVA) or the Chi-square test was used.

All variables which correlated with PP with a level of significance <0.01 in these analyses (given the relatively large sample size and multiple analyses) were included in a multivariable linear regression model. Because of the relatively large number of the variables entered in the final model, variables were entered in five blocks. First serum OPG alone was added; block B included age, sex, presence of diabetes, body mass index (BMI), the Charlson comorbidity index and cumulative end stage kidney disease (ESKD) vintage; the next block included the use of immunosuppressive medications: steroids, cyclosporine, tacrolimus, azathioprine, mycophenolate-mofetil and sirolimus; block D included eGFR, serum albumin, glucose and cholesterol, the final block included serum calcium, phosphorus, PTH, the use of active vitamin D and the use of phosphate (PO4) binders. All variables were retained in the final model. Non-linear association between OPG versus PP was tested using the squared OPG term in the regression model and restricted cubic spline. Variance influence factors (VIF) were used to indicate collinearity between independent variables in the multivariate regression model. For all analysis, two-sided p values are reported and results have been considered statistically significant if p < 0.05.

Data were analyzed using the IBM SSPS Statistics for Macintosh 22.0 software (IBM^®^ Corp. Armonk, NY) and STATA MP version 13.1 (STATA Corp., College Station, TX).

## Results

Baseline characteristics of the sample are shown in Table 1. Mean age of the participants was 51 ± 13 years, 57% were male, 21% diabetic and 94% had hypertension. Median transplant vintage was 77 months (IQR: 41–121). Most of the patients were on steroids, mycophenolate mofetil and calcineurin inhibitors for immune suppression (see Table 1). The etiology of the underlying kidney disease was chronic glomerulonephritis in 23%, autosomal polycystic kidney disease in 18%, tubulointerstitial nephropathies in 13%, diabetic nephropathy in 5%, hypertensive nephropathy in 7%, other or unknown renal disease in 34% of patients. Antihypertensive medication administered included beta blockers in 717 (74%) patients, ACE inhibitors or angiotensin receptor blockers (ARB) in 288 (30%), calcium channel blockers in 537 (55%) individuals and diuretics in 323 (33%).

**Figure 1:**
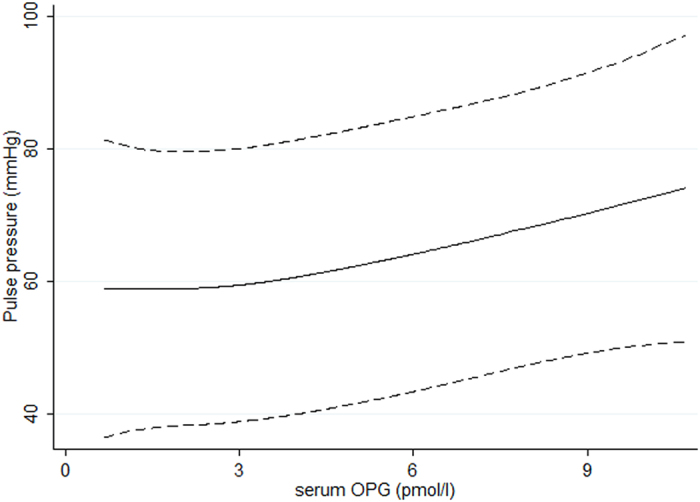
Non-linear association between OPG versus PP using restricted cubic spline. The model was adjusted according to model E in Table 3.

Associations between serum OPG tertiles versus several clinical and laboratory variables are also demonstrated in Table 1. Importantly, PP increased significantly with higher OPG tertiles (p < 0.001 for trend) and PP was significantly correlated with serum OPG levels (R = 0.284; p < 0.001) (Table 2 and Fig. 1). This association was significant in both younger and older individuals (below and above the mean age of the sample), among participants with preserved and more impaired eGFR (above and below of the mean eGFR of the sample), in patients with higher and more controlled blood pressure (above and below the mean systolic BP), among males and females and also in diabetics and non-diabetics (not shown).

Patients in the higher OPG tertiles were significantly older, were less likely male but more likely diabetic, and had somewhat lower BMI. The utilization of several immunosuppressive medications was also associated with OPG tertiles (Table 1). Patients in higher OPG tertiles had longer ESRD duration and lower eGFR, lower hemoglobin and serum albumin but higher phosphorus, PTH and alkaline phosphatase concentrations and higher Ca x P product. Finally, higher OPG tertiles were asociated with more comorbidities (Table 1). The association between serum OPG versus the mentioned variables was also demonstrated in correlation analysis (Table 2).

We also analyzed correlations between PP and demographic and clinical variables. PP was higher in males versus females (59 ± 16 vs 56 ± 17, p = 0.006) among patients with diabetes vs non-diabetics (65 ± 19 vs 56 ± 16, p < 0.001). PP was also significantly correlated with age, body mass index, hemoglobin level, total serum cholesterol, eGFR, serum albumin and serum glucose levels and the Charlson Comorbidity Index (Table 2).

To test if the association between OPG versus PP is independent of the potentially confounding covariables we built a multivariable linear regression model with PP as the dependent variable. As described in the Methods section, independent variables were entered in five blocks. Importantly, OPG was independently associated with PP in the fully adjusted final model (R^2^ = 0.233, beta = 0.143; p < 0.001) (Table 3). Additional independent predictors of PP in this model were age, sex, the Charlson Comorbidity Index, the presence of diabetes, use of cyclosporine A, and serum calcium (Table 4).

## Discussion

Here we demonstrated for the first time, in a large prevalent cohort of KTX patients that serum OPG levels were independently associated with pulse pressure, a surrogate marker of arterial stiffness and a cardiovascular risk factor. These results are compatible with the potential contribution of osteoprotegerin and also of CKD-MBD to increased CV risk in KTX recipients.

PP increases with declining eGFR in patients with CKD. It is also associated with age, the presence of diabetes mellitus and hypertension, serum phosphorus and PTH levels in this patient population^40^. Furthermore, PP appears to be significantly associated with age, sex, diabetes and dialysis vintage in hemodialysis patients after adjustment for systolic blood pressure^12^. Fernandez-Fresnedo *et al.* found that higher PP was associated with recipient age, systolic and diastolic blood pressure, presence of diabetes and cardiovascular disease in KTX recipients^41^. In our study PP was significantly associated with age and the presence of diabetes, consistent with previously published literature. Besides these factors, PP was also associated with cumulative ESRD time and eGFR, suggesting that kidney transplantation can not restore vascular lesions caused by prolonged uremia. Furthermore, progressive decline of the renal function due to chronic allograft injury could also play a role in determining arterial changes in these patients.

Elevated pulse pressure is a marker of arterial stiffness and is clearly associated with adverse cardiovascular outcomes^42^,^43^. In patients with CKD vascular calcification is thought to importantly contribute to vascular stiffness. Although the exact molecular mechanism of accelerated vascular calcification seen in patients with CKD is not fully understood, it has been repeatedly demonstrated that regulators of bone a mineral metabolism play a role in this pathophysiological process^7^,^44^,^45^. One of those factors is osteoprotegerin, a known inhibitor of osteoclastogenesis and bone resorption^46^. Interestingly, several animal models suggested a protective role for OPG against vascular calcification. Selective deletion of OPG in mice results in early-onset severe osteoporosis as well as significant medial calcification of the aorta and renal arteries^47^. In animal models OPG seems to prevent vascular calcification but is unable to reverse lesions once calcification has occurred^48^,^49^. Contrary to the apparent protective role of OPG observed in the animal models, higher serum levels of OPG are reportedly associated with increased vascular calcifications both in the general population and among patients with varying degree of CKD^22^,^23^,^25^,^26^,^27^,^30^. The reason for this paradoxical phenomenon is not fully explained. One potential hypothesis suggests that the elevated OPG levels may reflect a potential compensatory or protective reaction against procalcification factors^50^. It is also possible that OPG is a biomarker of the active calcification process in the vasculature. OPG, which is normally secreted by osteoblasts, may also be secreted by the osteoblast-like cells formed by the trans-differentiation of vascular smooth muscle cells. These cells express bone-specific proteins and actively deposit bone matrix in the vascular wall^51^.

Regardless of the yet unclear mechanistic role of OPG in vascular calcification, the clinical relevance of OPG lies in its prognostic potential. Serum OPG consistently predicted the presence and severity of coronary artery disease or mortality^6^,^26^,^27^,^29^,^34^,^52^,^53^. OPG levels decrease soon after kidney transplantation^31^ but post-transplant OPG levels are still associated with mortality in KTX patients^34^,^35^. Our results, demonstrating a consistent and independent association between serum OPG and pulse pressure, a surrogate of vascular stiffness, are consistent with the hypothesis that higher OPG level is a marker of vascular calcification and arterial stiffness. This is further corroborated by our finding of significant correlations between OPG versus markers of mineral metabolism (serum Ca, phosphorus and PTH) in our KTX sample, similarly to patients with CKD^29^ and on dialysis^25^.

In this study we wanted to assess the association between PP and OPG. We considered PP primarily as a surrogate of vascular stiffness. However, there is a complex relationship between PP and systolic and diastolic BP (SBP and DBP, respectively) and their predictive association with cardiovascular outcomes. Initially it had been thought that DBP was the most damaging component of BP. Subsequent epidemiologic studies, however, called attention to the relevance of SBP over DBP in inducing cardiovascular and renal damage^54^. It is well established that DBP decreases after about 50–55 years of age in the general population, whereas SBP progressively increases up to at least 80 years of age^55^. These relationships, however, may be different in patients with CKD, in whom atherosclerosis and arterial calcification is accelerated and systolic hypertension is very prevalent. Recently it has been shown that OPG levels correlated with systolic BP^56^,^57^. The underlying mechanism for that association was not quite clear but it was suggested that it may be related to vascular calcification, atherosclerosis or altered bone metabolism. In our dataset OPG was significantly correlated with both systolic and diastolic BP, in addition to PP. Furthermore, when we normalized PP to systolic BP, as recently suggested, the significant associations remained. The association between OPG and PP, therefore, likely reflect the underlying link between OPG and vascular calcification – although we cannot conclude to the directionality of this association from our data.

Several limitations of our study should be considered when interpreting our results. Patients from a single center were enrolled; therefore our results are not to be generalized without further considerations. Patients who were not participating in the study may have been different from participants, which is a potential source of bias. We think, however, that it is unlikely that this would have qualitatively changed our results. Only Caucasian patients participated which may make comparisons with multiethnic populations difficult. Moreover, our patients form a heterogenic group selected at various moments in their evolution after renal transplantation and with various degree of kidney dysfunction so that OPG levels varies. Finally, we did not measure vascular stiffness in this research that would have provided additional new information about vascular characteristics.

In conclusion we have demonstrated that OPG is independently associated with pulse pressure in renal transplant patients. These results support the potential role of disordered bone and mineral metabolism in vascular calcifications and stiffness in kidney transplant recipients. Prospective studies will be necessary to demonstrate the pathophysiologic link between disordered bone and mineral metabolism and vascular calcification in KTX patients. Those studies will need to be informed by basic research generating new knowledge using cell culture experiments and animal models. They will need to demonstrate a prospective association between changing bone metabolism and increasing vascular stiffness/calcification by utilizing multiple biomarkers of bone metabolism. This may be complemented by dual-photon x-ray absorptiometry or quantitative CT assessment of bone mineral density and structure. Vascular calcification will need to be determined by appropriate imaging, preferably CT scanning of coronaries and/or the aorta. This needs to be complemented by validated measurement of vascular stiffness. In addition to these studies, potential intervention trials that aim at modulating bone turnover, may demonstrate the effect (or the lack thereof) on vascular calcification of interventions that modify bone turnover.

## Additional Information

**How to cite this article**: Nemeth, Z. K. *et al.* Serum osteoprotegerin is associated with pulse pressure in kidney transplant recipients. *Sci. Rep.* **5**, 14518; doi: 10.1038/srep14518 (2015).

## Figures and Tables

**Table 1:** Demographic and biochemical data for all participants and for tertiles of serum OPG (data are presented as mean ± standard deviation (SD), median with interquartile range (IQR) or n[%] as appropriate).

	Total sample(n = 969)	Tertiles of serum OPG (pmol/L)			p fortrend
		1^st^(<3.20)	2^nd^(3.20–4.39)	3^rd^(>4.39)	
Pulse pressure (mmHg)	58 ± 17	53 ± 14	57 ± 17	63 ± 18	<0.001
Age (years)	51 ± 13	44 ± 13	51 ± 12	57 ± 10	<0.001
Sex (male)	559 (57)	198 (63)	191 (58)	173 (52)	0.02
Charlson Comorbidity Index	2 (2)	2 (1)	2 (2)	3 (2)	<0.001
Diabetes mellitus (yes)	206 (21)	39 (12)	78 (24)	89 (27)	<0.001
BMI (kg/m^2^)	26.9 ± 4.8	27.4 ± 5	27.1 ± 4.8	26.5 ± 4.7	0.015
Tx vintage (months)	72 (75)	64.5 (82)	76 (74)	76 (71)	0.022
ESKD vintage (months)	107.5 (87)	100 (90)	108 (89)	113 (93)	0.003
Systolic BP (mmHg)	142 ± 19	138 ± 18	141 ± 19	147 ± 20	<0.001
Diastolic BP (mmHg)	84 ± 12	85 ± 12	83 ± 12	83 ± 12	0.073
Steroids (yes)	787 (81)	255 (78)	266 (81)	277 (85)	0.070
CSA (yes)	469 (48)	145 (44)	160 (49)	172 (53)	0.088
Tacrolimus (yes)	418 (43)	164 (50)	136 (42)	122 (37)	0.003
MMF (yes)	755 (78)	269 (82)	257 (79)	240 (73)	0.029
Sirolimus (yes)	78 (8)	16 (5)	23 (7)	39 (12)	0.003
CKD-EPI eGFR (ml/min/1.73 m^2^)	53 ± 22	60 ± 21	54 ± 20	44 ± 22	<0.001
Albumin (g/l)	40 ± 4	41 ± 4	40 ± 4	39 ± 4	<0.001
Blood glucose (mmol/l)	6.6 ± 2.6	6.3 ± 2.4	6.8 ± 2.8	6.6 ± 2.4	0.037
CRP (mg/l)	3.1 (5.3)	2.9 (4.7)	3.4 (5.4)	3.2 (5.7)	0.25
Cholesterol (mmol/l)	5.51 ± 1.27	5.36 ± 1.14	5.63 ± 1.32	5.54 ± 1.34	0.069
Trigliceride (mmol/l)	2.08 (1.29)	1.71 (1.42)	1.69 (1.31)	1.68 (1.19)	0.917
Phosphorus (mmol/l)	1.1 ± 0.3	1.1 ± 0.3	1.1 ± 0.2	1.2 ± 0.3	0.001
Ca (mmol/l)	2.3 ± 0.2	2.3 ± 0.1	2.3 ± 0.1	2.3 ± 0.1	0.427
Ca*P (mmol^2^/l^2^)	2.5 ± 0.6	2.4 ± 0.7	2.4 ± 0.5	2.6 ± 0.6	<0.001
PTH (pg/ml)	67 (56)	62 (47)	69 (54)	75 (75)	<0.001
Alkaline phosphatase(UI/l)	88 ± 40	85 ± 31	86 ± 34	94 ± 50	0.005
Osteoprotegerin(pmol/l)	3.9 ± 1.5	2.4 ± 0.53	3.7 ± 0.35	5.6 ± 0.11	<0.001

**Table 2:** Correlation between PP and OPG versus clinical and laboratory variables.

	PP (mmHg)	Osteoprotegerin(pmol/l)
Osteoprotegerin (pmol/l)	R = 0.284	NA
	p < 0.001	
Pulse pressure (mmHg)	NA	R = 0.284
		p < 0.001
Age (years)	R = 0.385	R = 0.419
	p < 0.001	p < 0.001
Charlson Comorbidity Index	R = 0.232	R = 0.250
	p < 0.001	p < 0.001
BMI (kg/m^2^)	R = 0.133	R = −0.079
	p < 0. 001	p = 0.014
Tx vintage (months)	R = −0.006	R = 0.087
	p = 0.845	p = 0.006
Cumulative ESKD vintage (months)	R = −0.023	R = 0.107
	p = 0.482	p = 0.001
eGFR	R = −0.105	R = −0.290
	P < 0.001	P < 0.001
Hemoglobin (g/l)	R = −0.095	R = −0.160
	p = 0.003	p < 0.001
CRP (mg/l)	R = 0.072	R = 0.023
	p = 0.025	p = 0.465
se Albumin (g/l)	R = −0.134	R = −0.230
	p < 0.001	p < 0.001
se Cholesterol (mmol/l)	R = 0.094	R = 0.041
	p = 0.003	p = 0.199
se Glucose (mmol/l)	R = 0.172	R = 0.037
	p < 0.001	p = 0.244
se Calcium (corrected for albumin) (mmol/l)	R = −0.122	R = −0.104
	p < 0.001	p = 0.001
se Phosphorus (mmol/l)	R = 0.031	R = 0.144
	p = 0.328	p < 0.001
Ca x P (mmol^2^/l^2^)	R = −0.022	R = 0.145
	p = 498	p < 0.001
Se ALP (U/l)	R = 0.002	R = 0.107
	p = 0.948	p = 0.001
Se PTH (pg/ml)	R = 0.016	R = 0.075
	p = 0.616	p = 0.019

**Table 3:** Multivariable linear regression model of pulse pressure as dependent variable to assess the independent association with serum OPG.

	serum OPG		
	beta	P Value	R square
model A	0.284	<0.001	0.081
model B	0.152	<0.001	0.209
model C	0.152	<0.001	0.216
model D	0.141	<0.001	0.224
model E	0.143	<0.001	0.233

Shown in the cells are the parameters of the independent variable serum OPG.

Independent variables entered into the model: **Block A**: serum OPG alone; **Block B**: Block A + age, sex, presence of diabetes, BMI, the Charlson Comorbidity Index and cumulative ESKD vintage; **Block C**: Block B + use of immunosuppressive medications (steroids, cyclosporine, tacrolimus, azathioprine, mycophenolate-mofetil and sirolimus); **Block D**: Block C + eGFR, serum albumin, glucose and cholesterine; **Block E**: Block D + calcium, phosphorus, PTH, use of active vitamin D, use of PO4 binders. Abbreviations: OPG – Osteoprotegerin.

**Table 4:** Multivariable regression analysis for PP (R^2^ = 0.233, p < 0.001). Shown in the table are variables which were independently associated with PP.

	B	StandardizedCoefficients	p value	95% ConfidenceInterval for B	
		Beta		LowerBound	UpperBound
Se OPG	1.636	0.143	<0.001	0.865	2.407
Age	0.342	0.259	<0.001	0.251	0.434
Gender	−3.968	−0.116	<0.001	−6.006	−1.930
Presence of diabetes	2.874	0.070	0.044	0.080	5.668
Charlson Comorbidity Index	0.913	0.093	0.005	0.271	1.555
Cumulative ESKD vintage	−0.016	−0.062	0.053	−0.033	0.000
Taking Cyclosporin A	3.749	0.111	0.047	0.047	7.451
Se Ca	−10.361	−0.094	0.004	−17.400	−3.321

The final model was also adjusted for the following variables: BMI, use of steroids, tacrolimus, azathioprine, sirolimus and mycophenolate-mofetil, eGFR, se Albumin, phosphorus, glucose and PTH.
